# High-Efficiency of Bi-Functional-Based Perovskite Nanocomposite for Oxygen Evolution and Oxygen Reduction Reaction: An Overview

**DOI:** 10.3390/ma14112976

**Published:** 2021-05-31

**Authors:** Tse-Wei Chen, Palraj Kalimuthu, Ganesan Anushya, Shen-Ming Chen, Rasu Ramachandran, Vinitha Mariyappan, Durai Chidambaranathan Muthumala

**Affiliations:** 1 Department of Materials, Imperial College London, London SW7 2AZ, UK; t.chen19@imperial.ac.uk; 2 School of Chemistry and Molecular Biosciences, University of Queensland, Brisbane 4072, Australia; p.kalimuthu@uq.edu.au; 3 Department of Physics, S.A.V. Sahaya Thai Arts and Science (Women) College, Sahayam Nagar, Kumarapuram Road, Vadakkankulam, Tirunelveli 627116, India; anushya@savsahayathaicollege.com; 4 Electroanalysis and Bioelectrochemistry Lab, Department of Chemical Engineering and Biotechnology, National Taipei, University of Technology, No.1, Section 3, Chung-Hsiao East Road, Taipei 106, Taiwan; vinithavicky80@gmail.com; 5 Department of Chemistry, The Madura College, Vidya Nagar, Madurai 625011, India; 6 Department of Chemistry, Sri Kumara Gurupara Swamigal Arts College, Thoothukudi 628619, India; dmuthumala@gmail.com

**Keywords:** bi-functional perovskite, synthesis route, nanocomposite, oxygen reduction reaction, oxygen evolution reaction

## Abstract

High efficient, low-cost and environmentally friendly-natured bi-functional-based perovskite electrode catalysts (BFPEC) are receiving increasing attention for oxygen reduction/oxygen evolution reaction (ORR/OER), playing an important role in the electrochemical energy conversion process using fuel cells and rechargeable batteries. Herein, we highlighted the different kinds of synthesis routes, morphological studies and electrode catalysts with A-site and B-site substitution co-substitution, generating oxygen vacancies studies for boosting ORR and OER activities. However, perovskite is a novel type of oxide family, which shows the state-of-art electrocatalytic performances in energy storage device applications. In this review article, we go through different types of BFPECs that have received massive appreciation and various strategies to promote their electrocatalytic activities (ORR/OER). Based on these various properties and their applications of BFPEC for ORR/OER, the general mechanism, catalytic performance and future outlook of these electrode catalysts have also been discussed.

## 1. Introduction

**Scheme 1:**
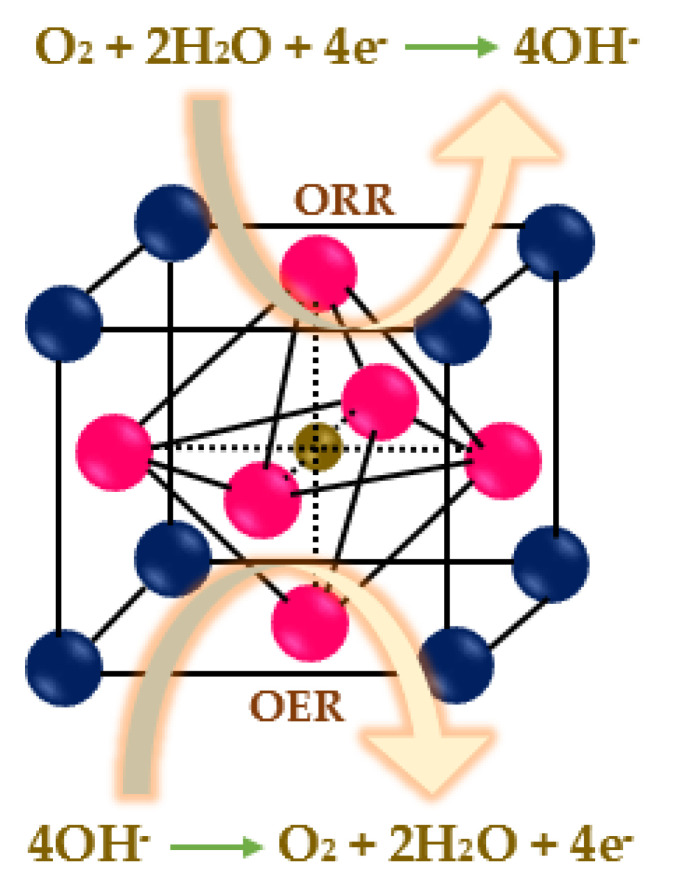
Mechanism of oxygen reduction reaction (ORR) and oxygen evolution reaction (OER) in alkaline medium.

Nowadays, most human beings are consuming energy from various renewal sources that have widespread applications in human activities, correspondingly conversion of chemical energy into electrical energies, like supercapacitors [1], fuel cells [2], batteries [3], solar cells [4] and microbial fuel cells [5], etc. The sustainable regenerative energies are obtained from the fundamental electrochemical concepts (energy conversion/storage) of both oxygen reduction and oxygen evolution reactions [6]. Most of the researchers have focused on the development of major scientific and technology-oriented inexpensive, efficient, noble metal-free and stable electrode catalysts, which can be used as next-generation sustainable energy storage devices and can improve their electrocatalytic energy storage activities [7]. Since the 1980s, the cubic crystal structure of ABO_3_-type perovskites has received attention as themost promising electrode candidate for highly efficient electrocatalysts in both ORR and ORE, because of cost-effectiveness, fascinating electrochemical properties, high-power density, high-energy-density and long-term cyclic durability in electric vehicles [8,9,10,11]. Herein, an interesting bi-functional oriented Ba_0.5_Sr_0.5_Co_0.8_Fe_0.2_O_3−δ_based g-C_3_N_4_-supported Vulcan carbon composite electrode catalyst also expedites an efficient suitable air cathode for the ORR and OER process [12]. The cost-effective electrode materials of double perovskite CaCu_3_Ti_4_O_12_ (CCTO) have gained significant attention due to lower overpotential, excellent electrocatalytic performances and high electrode stability for both ORR and OER reactions [13]. Furthermore, developing Fe-based Bi_0.5_Sr_0.5_Fe_0.95_Mo_0.05_O_3−δ_ perovskite electrodes have successfully been studied as a potential electrode candidate for practical uses in solid-oxide fuel cells (SOFCs). In addition to that, thermal expansion behavior, phase structure and electrochemical properties are also evaluated [14]. Recently, Vij and his co-workers [8] have reviewed nickel-based electrode catalysts, which display exciting electronic properties, better surface adsorption assets, high corrosive resistance with a synergetic effect that takes place between nickel and their neighbouring molecules for their clean energy applications. Rai et al. [15] have developed an inexpensive, efficient and bi-functional fluorinated-based copper-manganese oxide (FCMO) electrode catalyst, which was showed both ORR and OER under acid and alkaline conditions. The catalyst also exhibited excellent electrochemical reactions, and it was found to be a two-electron transfer process. The bi-functionality-natured nitrogen-doped carbon nanostructures (CNs) have been prepared by the ball-milling method. Thus, the ORR and OER electrocatalytic activity has been observed to increase in the pyridinic-N site and can be used as a rational catalyst [16]. La_0.8_Sr_0.2_Mn_0.95_Sc_0.25_Po_0.025_O_3−δ_ (LSMSP) is one of the most significant active and robust-perovskite-based electrode catalysts for superior ORR studies. The developed co-doping LSMSP perovskite oxide can play a crucial role in ORR activity and excellent cyclic durability under alkaline conditions [17]. On the other hand, Zhang et al. [18] have reported a novel perovskite composition of SrSc_0.175_Nb_0.025_Co_0.8_O_3−δ_ (SSNC) and Ba_0.5_Sr_0.5_Co_0.8_Fe_0.2_O_3−δ_ (BSCF) that can enable oxygen reduction reaction with the delivered power density value of910 mW cm^−2^. Accordingly, a non-precious metal (NPM)-based cobalt and N-doped multi-walled carbon nanotube (N-MWCNTs-Co) catalyst has been synthesized via solid-state pyrolysis (SSP) method, where the fabricated N-MWCNTs-Co catalyst can interact with hydrated alkali metal cations and adsorbed oxygen-based species for ORR and OER activities [19]. In particular, a novel cobalt-free-based perovskite (SrSc_0.025_Nb_0.075_Fe_0.9_O_3−δ_) composite has received special attention, because of its favorable structural arrangements and for the treatment of improved activity for ORR, which significantly enhances their CO_2_ resistivity [20]. Xu et al. [21] have fabricated tin and iron co-doped BaCo_0.9-x_Fe_x_Sn_0.1_O_3−δ_ (BCFSn) perovskite by a conventional solid-state reaction-based method, while the developed BCFSn perovskite displayed intrinsic OER activity under alkaline conditions with superior electrode OER stability. Recently, Kuai et al. [22] designed BaCo_0.4_Fe_0.4_Zr_0.4_Y_0.1_O_3−δ_ (BCFZY) cathode materials, which showed better phase stability, attractive performance at low-to-medium temperature conditions and significantly enhanced their ORR activity with the reported specific resistance value of 0.011 Ω cm^2^. Zhuang et al. [23] have prepared ultrathin iron-cobalt oxide (FexCoy-ONSs) nanosheets by a chemical method, based on abundant active sites and high surface area of the FexCoy-ONSs electrode catalyst leading to significantly enhanced their electrode conductivity and could improve the catalytic activity of OER. Table S1 compares the synthesis route, morphologies and supporting electrolytes for ORR and OER of different types of perovskite-based electrocatalysts. A brief schematic representation of an energy-based application is given in Scheme 1. The mechanism is mainly focused on perovskite-based electrode catalysts for ORR and OER.

In this article, we elaborated on the recent development of nanocomposite-based bi-functional perovskite electrode catalyst, which emphasizes the possible mechanism and electrochemical energy storage in both ORR and OER. Additionally, we discussed design, various synthesis approaches, different analytical and electrochemical characterizations, fabrication of nanocomposites and extensive studies of electrocatalyst based on perovskite-based composites. Extensive investigations are made to analyze their physical properties and the charge transfer between A (mono-valet metallic cation) and B (di-valent metallic cation), which could play an essential role in electrochemical applications. Furthermore, the designing of high-performance-based bi-functional-supported perovskite electrocatalyst highlighted better ORR and OER activities.

## 2. Synthesis of Perovskites

**Figure 1:**
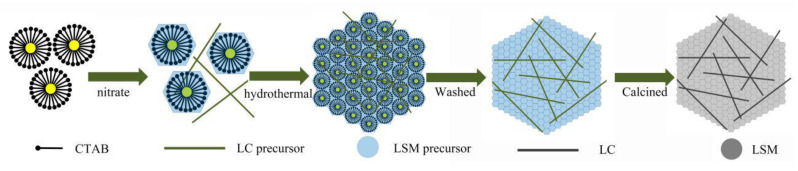
Synthesis of the LC-LSM hybrid catalyst. Copyright 2018 by the American Chemical Society [36].

Several methodologies have been established to synthesize perovskite oxides, including the conventional solid-state method [24], combustion synthesis [25], co-precipitation [26], hydrothermal/solvothermal methods [27], sol–gel [28] and polymer-assisted approach [29]. The solid-state reaction, combustion and co-precipitation methods, amongst others, necessitate extreme reaction temperature as well as other adverse unfavorable conditions like elevated pressure and special equipment. Furthermore, the synthesized substance generally has relatively large-sized particles (micron size) and a small surface area, which is not conducive to improving their electrocatalytic activities. Although the hydrothermal/solvothermal approach could be employed to synthesize perovskites with a variety of distinct surface structures, its efficiency is limited due to reactor size constraints. Consequently, the sol–gel and polymer-assisted methods were used to achieve perovskites for broad-scale manufacturing. While the sol–gel process requires more than one step, which initially converts the sol into gel followed by a calcination process, the polymer-assisted method only encompasses the thermal treatment of the metal–polymer complex solution to obtain the desired product, and it is also suitable for broad-scale production [30]. Additionally, several other methods for synthesizing perovskites have been devised. For instance, Chang et al. [31] have prepared La_0.6_Ca_0.4_CoIr_0.25_O_3.5−δ_ perovskite powders using a mechanical alloying process. The galvanometric measurements showed that the electrocatalytic stabilities of the prepared perovskite powders are stable and sustainable. Thermal decomposition of freeze-dried citrates and the Pechini process were used to obtain nickel and iron substituted LaCoO_3_ with rhombohedral distorted perovskite structures in the temperature range of 600–900 °C [32]. Moreover, a simple electrospinning technique coupled with successive calcination was used to synthesize Mg-doped perovskite LaNiO_3_ (LNO) nanofibers (LNMO NFs) [33]. The aforementioned synthesis methods necessitate a complex process as well as specialized equipment, which increases the possibility of material synthesis and the cost of the products. In this regard, a suitable and feasible synthesizing method needs to be chosen based on the perovskites applications [34]. The Hydrothermal/Solvothermal method pertains to the preparation of materials via chemical processes in solution at temperatures and pressures above ambient in a sealed setting [35]. La_2_O_2_CO_3_-La_0.7_Sr_0.3_MnO_3_ (LC-LSM) hybrid material was prepared via the hydrothermal method. The solvents strontium nitrate, manganese nitrate and lanthanum nitrate, cetyltrimethyl ammonium bromide in different molar ratios were dissolved in distilled water. The KOH solution was then added to the above mixture, and the pH of the solution was adjusted to 9. The solution was taken in a Teflon container. The hydrothermal tank was heated to 180 °C and maintained at the temperature for 2 h. The deposit was washed three times with deionized water and with ethanol. Eventually, the product was dried at 80 °C for 24 h and calcined at 500 °C for 2 h. Figure 1 shows the schematic representation for synthesizing the LC-LSM hybrid catalyst [36].

An ionic-liquid method was employed to synthesize highly crystalline Ba_0.5_Sr_0.5_CoxFe_1−x_O_3−δ_ with the whole compositional range, resulting in a high degree of phase purity [37]. Interestingly Du and his co-authors [38] have synthesized the nonstoichiometric CaMnO_3-__δ_using the Pechini route. The as-prepared perovskite material showed an enhanced electrical property. Moreover, it is a promising active, low-cost bifunctional catalytic material for reversible ORR and OER reactions. The sol–gel method entails converting a liquid to gel, followed by post-treatment to produce the solid substance. The key advantage of the sol–gel approach is that it can produce materials with excellent purity and uniform nanostructures. Accordingly, the sol–gel method has been extensively used in a variety of fields, including the preparation of catalysts, specifically perovskite oxides [39,40]. Liu et al. [41] reported the synthesis of dual perovskite Sr_2_CoMo_1−x_NixO_6-δ_ using the sol–gel method. Initially, Sr(NO_3_)_2_, Co(NO_3_)_2_·6H_2_O, (NH_4_)_6_Mo_7_O_24_·4H_2_O and Ni(NO_3_)_2_.6H_2_O were dissolved in distilled water in the appropriate molar ratios. In order to form a clear solution, it was made to stir vigorously on a hot plate at 200 °C. The EDTA and CA were then mixed into the above solution to acts as complexing agents. To prevent precipitation, the pH of the solution was set to about 7 using NH_3_·H_2_O. After completely removing the water from the solution through evaporation under thermal treatment, a transparent sol–gel was obtained, which was then transferred to a muffle furnace to be sintered in air at 600 °C for 4 h and then at 1100 °C to get the desired double perovskite oxide powder. Similarly, Fabbri et al. [42] studied the electrochemical activity and selectivity toward the oxygen reduction reaction for Ba_0.5_Sr_0.5_Co_0.8_Fe_0.2_O_3-__δ_composite electrode prepared via the sol–gel route. In the experimental procedure, Ba(NO_3_)_2_, Sr(NO_3_)_2_, Co(NO_3_)_2_·6H_2_O and Fe(NO_3_)_2_ were dissolved in nitric acid solution. Citric acid was used as a chelating agent. The pH of the resultant transparent solution was adjusted between 6 and 8 by NH_4_OH solutions. After that, thermal treatment was used to extract the water content before it converted into a gel and was then ignited to produce black ash. Cao et al. [43] reported the synthesis of Cu_2_ZnSnS_4_ nanoparticles via a hot injection technique. In this technique, the authors used CZTS nanoparticle ink to prepare a hole transporting layer in a paintable carbon electrode. Furthermore, the spin-coating speed and annealing time of the CZTS hole transporting layer are optimized sequentially. Perovskite type barium titanate nanoparticles decorated on RGO (reduced graphene oxide) nanosheets were synthesized successfully using the sonochemical process for determining ractopamine in meat samples [44]. La_4_BaCu_5_O_13+__δ_ and La_6.4_Sr_1.6_Cu_8_O_20__±δ_ perovskites have been synthesized by a Pechini method. The synthesized perovskite materials exhibit good electrical conductivity and have been explored as promising cathode materials for IT-SOFC applications [45]. Perovskites can be known as a compound made up of two metal oxides. Consequently, perovskites are typically synthesized using the solid-state approach, wherein two metal oxides are combined and incorporated through high-temperature calcination. For instance, perovskite NdBaMn_2_O_5_ has been synthesized by the solid-state method in decreasing the atmosphere. Moreover, this material was considered a fascinating material for anode and cathode symmetric SOFC [46].

## 3. Morphology of Perovskites

**Figure 2:**
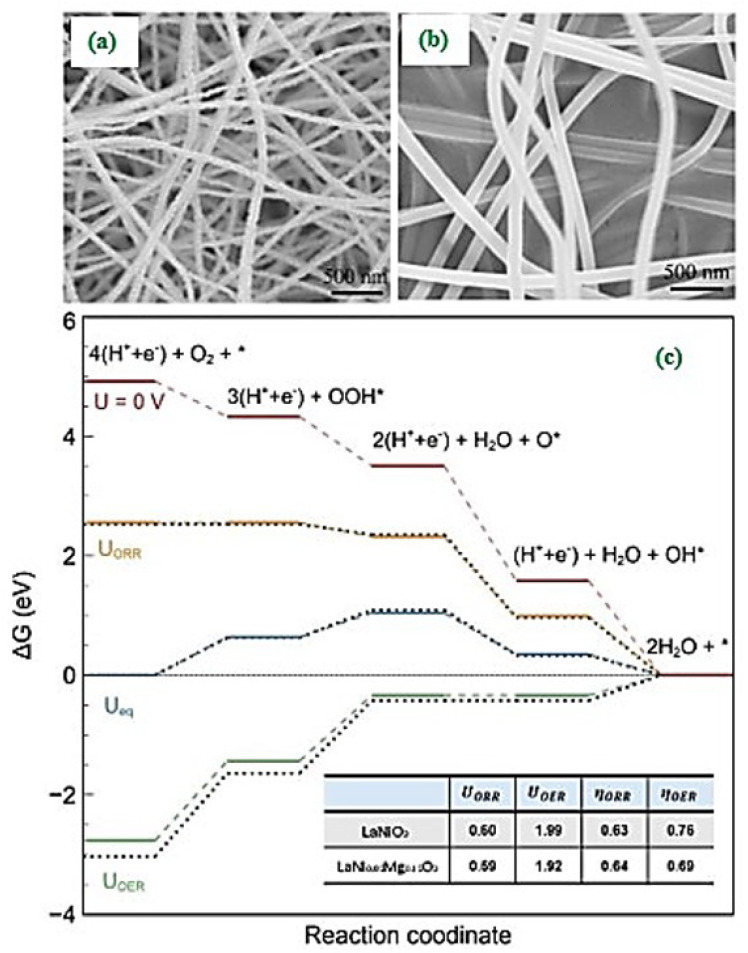
(**a**) SEM image of the as-electrospun product of LNMO (**b**) SEM image of the LNMO NFs after calcination at 700 °C (**c**) Computed reaction free energy diagram for ORR/OER reactions on LNMO 001 surfaces. Copyright 2019 by the American Chemical Society [33].

Perovskite materials have received much interest because of their power efficiency, ease of fabrication, low cost and other photovoltaic applications. The morphology of the perovskites is very important when it comes to applications. In this section, we discussed various perovskite materials and their morphological structures from a different perspective. LaNiO_3_ and LaNi_0.85_Mg_0.15_O_3_ nanofibers were synthesized via an electrospinning method. The prepared LNMO NFs exhibit superior catalytic activity over OER and ORR than LaNiO_3,_ and also it had a more positive half-wave potential (0.63 V) and lower overpotential of 0.45 V at a current density of 10 mA cm^−2^. As shown in Figure 2, the morphology of the LNMO has remained unchanged after calcination (Figure 2a), and the LNMO NFs have shrunk slightly (Figure 2b) [33]. Since the LNMO has a strong surface oxygen-binding that increases OER activity (Figure 2c), it can be used as a promising bifunctional catalyst for Zinc–air battery applications. A novel composite cathode PrSrFe_0.5_Co_0.5_O_4_−Pr_0.4_Sr_0.6_Fe_0.5_Co_0.5_O_3_ (PSFC_214-113_) was synthesized by a sol–gel method. The voltage output of the composite cathode at a steady current load demonstrated that the cell voltage remained stable over time, verifying the excellent ORR stability of the PSFC_214-113_. In addition, the intensity of the spent materials is lower than that of the fresh sample. The cross-sectional scanning electron micrographs reveal no substantial SrO after stability measurement. Additionally, the electrode retains its original morphology and only partial agglomeration after stability testing [47].

**Figure 3:**
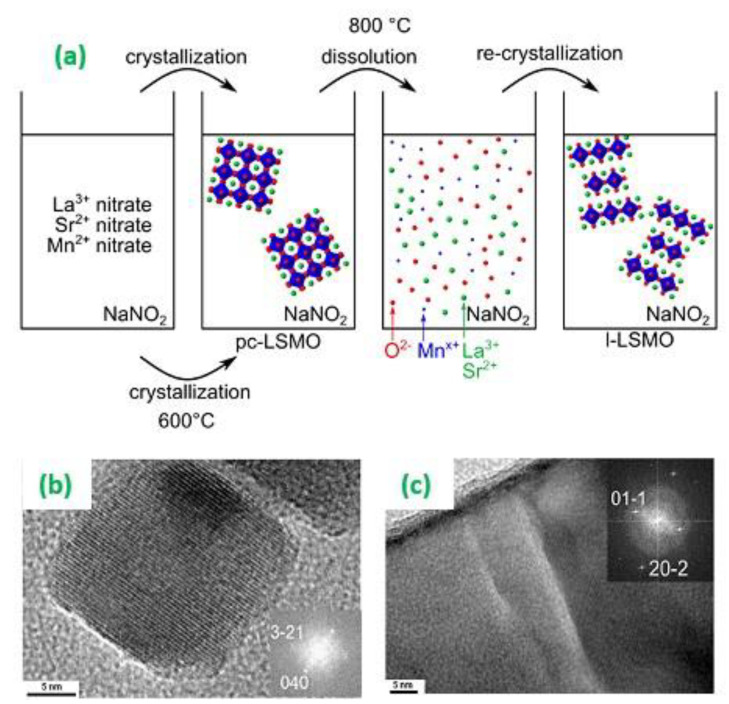
(**a**) Reaction pathway yielding layered perovskite (l-LSMO) nanoparticles from pc-LSMO into molten NaNO_2_, (**b**) HRTEM images of pc-LSMO nanocubes and (**c**) HRTEM image of an l-LSMO nanoparticle and corresponding fast Fourier transform.Copyright 2020 by the American Chemical Society [49].

Perovskite-structured calcium and strontium-doped rare-earth cobaltate powders were synthesized by various processes. The powders prepared have a cubic or rhombohedral structure form of different morphologies [48]. The new types of ligand-free powdersbased on two different layered (La_0.5_Sr_1.5_MnO_4_, pc-LSMO) and pseudocubic (La_0.7_Sr_0.3_MnO_3_, I-LSMO) perovskites were synthesized by one-pot route (Figure 3a). Among these nanocubes (Figure 3b) and nanocrystals (Figure 3c), pc-LSMO nanocrystals could exhibit better ORR activity (21.4 A g^−1^) with remarkable cyclic stability under alkaline conditions [49]. Ruthenium-based pyrochlores (A_2_Ru_2_O_7_, A=Y, Nd, Bi) composite has shown the distribution of nanoparticles, which is analyzed by STEM-EDX analysis and the systematic theoretical study of OER activities [50].

**Figure 4:**
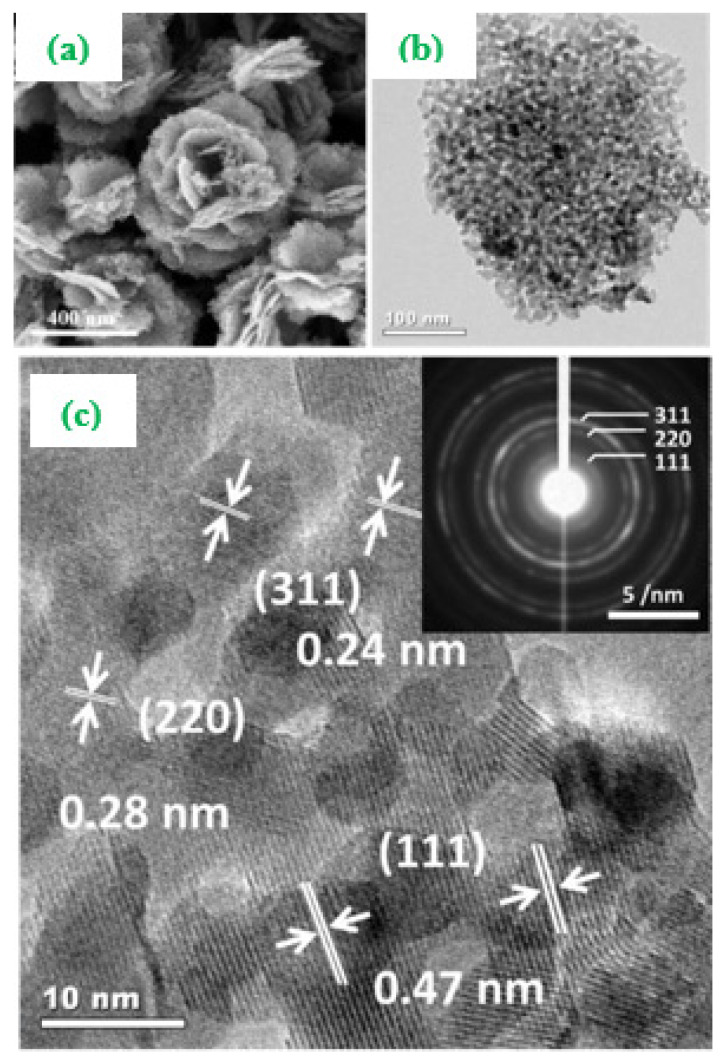
(**a**) SEM (**b**) TEM (**c**) HR-TEM images with an inset of SAED pattern of 20% Mn-Co. Copyright 2016 by the American Chemical Society [52].

LaNiO_3_, La_2_NiO_4_, and SrLaNiO_4_ nanoparticles were synthesized through the molten salts synthesis method. According to Scanning Electron Microscopy (SEM) and Transmission Electron Microscopy (TEM), LaNiO_3_ is made up of well faceted truncated cubes with an average particle size of 100 nm. Tiny particles as small as 20 nm can also be seen in these micrographs [51]. Song et al. [52] synthesized Ni/Mn promoted cobaltite by a one-step wet-chemical method. The inclusion of Mn might pull apart the microspheres and nanosheets, prohibiting Meso-Co nanoparticles from aggregating, and this is consistent with the SEM results (Figure 4a,b). Moreover, as the Mn content increases, the X% Mn-Co content decreases resulting in an increase in surface area. The lattice fringes in the HR-TEM image and the SAED ring patterns in the figure confirm the good crystallinity of the prepared mesoporous materials (Figure 4c).

La_0.6_Sr_0.4_Co_0.2_Fe_0.8_O_3−δ_ (LSCF) perovskites have been synthesized by a wet chemical process. Field Emission Scanning Electron Microscopy (Fe-SEM) reveals that the LSCF perovskites have a smooth surface. When the weight percentage of Pd increases to 30%, Pd particles entirely cover the surface of LSCF [53]. Similarly, the PrBa_0.94_Co_2_O_5+__δ_ sample was prepared by a solid-state reaction method. SEM micrographs reveal that the particles are embellished with a few nanorods. The precursor PBC_−1_, on the other hand, is comprised of irregular particles ranging in size from 0.5 to 1.5 µm [54].

## 4. Electrochemical Impedance Spectroscopy (EIS)

**Figure 5:**
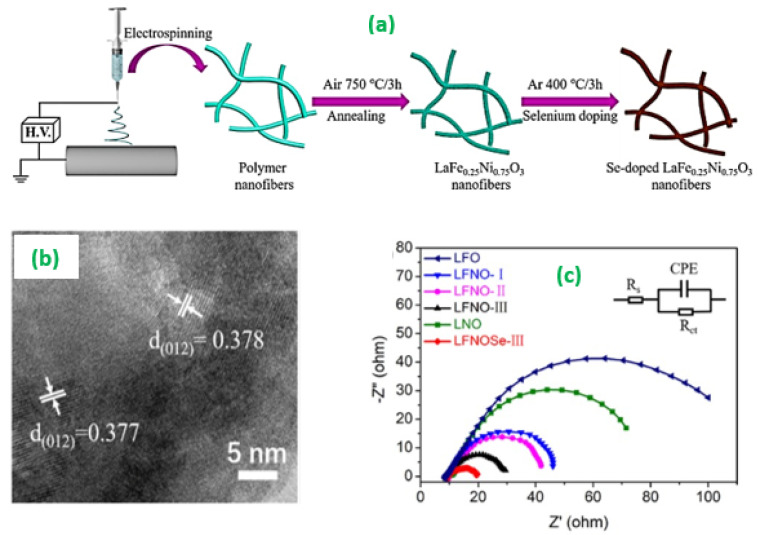
(**a**) shows the schematic illustration of the processing of Se-doped LaFe_0.25_Ni_0.75_O_3_ nanofibers, (**b**) HRTEM image of LFNOSe-III and (**c**) Nyquist plots of the as-prepared catalysts of LFNOSe-III. Copyright 2020 by the American Chemical Society [57].

Electrochemical impedance spectroscopy (EIS) denotes a method that is used to acquire insight into the bulk and interfacial properties of multi-junction devices, it is being used to analyze the devices under various in-situ conditions such as a function of dc voltage, illumination intensity and temperature. Over decades, it has been used by several researchers to study the response of perovskite solar cells under different operating temperatures. This EIS technique provides the impedance spectra of the samples against a frequency range of 0.1–1 MHz against an AC voltage. It is also interpreted as a Nyquist plot with Z real and Z imaginary along the *x*-axis and *y*-axis, respectively [55]. This EIS analysis has been used for the study of electrochemical/structural properties of heterogeneous systems, like porous membranes and different kinds of modified nanocomposite electrodes. Additionally, it can measure the polarization resistance (low-frequency) and solution resistance (high-frequency), respectively [56]. Porous-based one-dimensional LaFe_1−x_Ni_x_O_3_ (LFNO NFs) perovskite nanofiber was prepared by a feasible electrospinning technique (Figure 5a), which generally enables one to measure interplanar distance (0.377 and 0.378 nm) by HR-TEM analysis (Figure 5b). From the Nyquist plot studies, LFNO NFs-III catalyst could exhibit lower charge–transfer resistance (R_CT_) value and improve its OER activities (Figure 5c) [57].

**Figure 6:**
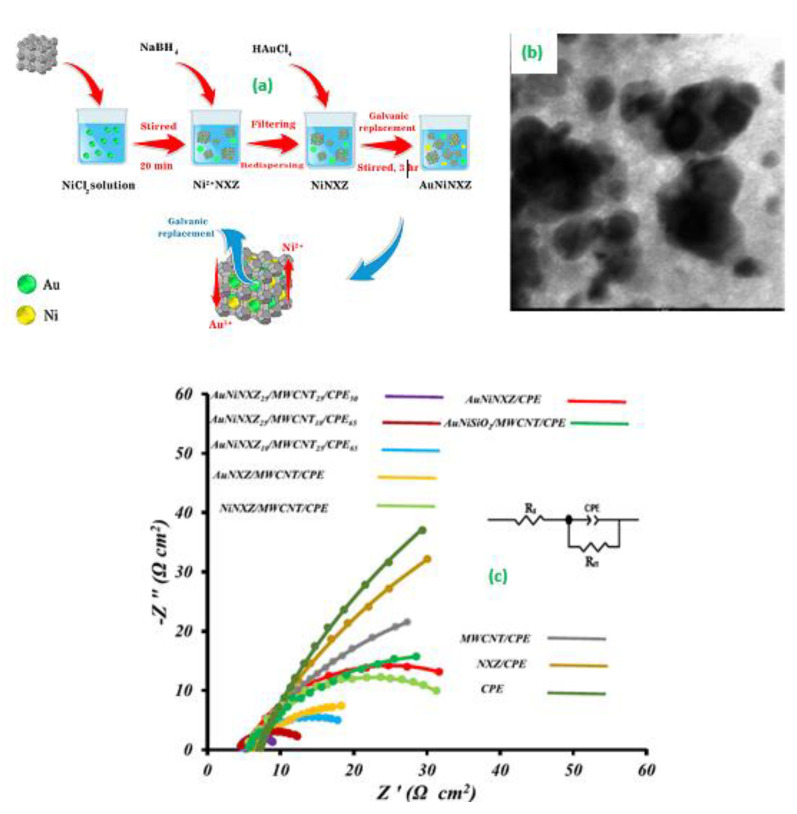
(**a**) Schematic Illustration of the AuNiNXZ Composite Preparation, (**b**) TEM images of AuNiNXZ and (**c**) Nyquist plot for different electrodes. Copyright 2019 by the American Chemical Society [58].

**Figure 7:**
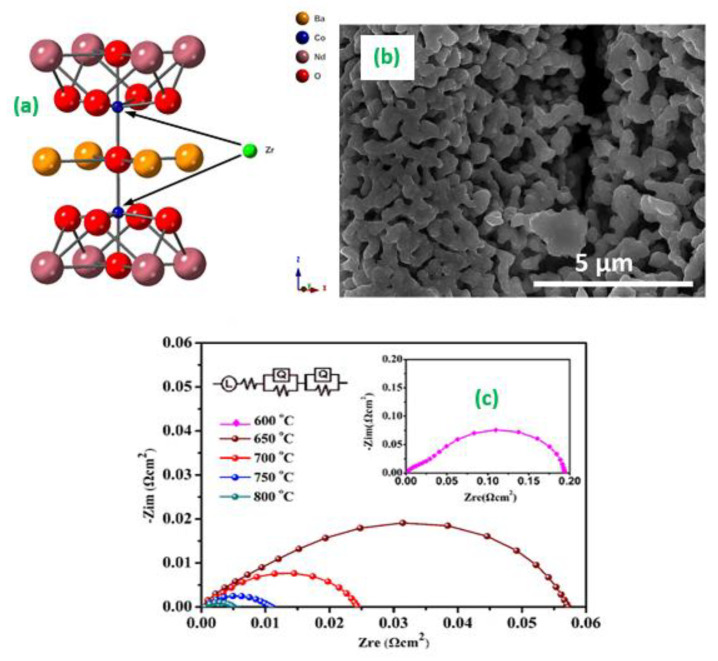
(**a**) crystal structure, and (**b**) SEM images of the microstructure for NdBaCo_1.95_Zr_0.05_O_5+δ_ and (**c**) Typical impedance spectra of symmetric cells measured under an open-circuit condition. Copyright 2019 by the American Chemical Society [59].

Amiripour et al. [58] prepared an inexpensive, novel and highly active AuNi bimetallic metal modified on a nano X zeolite (AuNiNXZ) composite considered as the green method (Figure 6a). The nonhomogeneous surface structured bimetallic nanoparticles information was received from TEM analysis (Figure 6b)and it can be recognized as a potential electrode catalyst, which can improve their electrocatalytic activity with favorable low overpotential (210 mV@10 mA cm^−2^) for OER (Figure 6c). The symmetrical study of Zr-doped NdBaCo_1.95_Zr_0.05_O_5+δ_ double perovskite (Figure 7a) electrode catalyst resulted in attractive cathode materials, good chemical compatibility with high SOFC conversion efficiency. From the scanning electron microscope (SEM) analysis, the unique morphological lattice structure, particle size and cathode porosity for NBCZrOare shown inFigure 7b. The typical EIS analysis can exhibit lower ASR values and the polarization impedance values can be reached at 0.006, 0.012, 0.024, 0.057 and 0.189 Ω cm^−2^ (applied temperatures 800, 750, 700, 650 and 600 °C), which indicates high ORR activity (Figure 7c) [59].

Interestingly, the crystal structure of cobalt-free BaFeO_3−δ_ (BF) perovskite has become an innovative new class of electrode catalyst, specifically the surface-active cathode materials that play an important role in the development of oxygen-ion conductivity for SOFCs and exhibit a power density value of 870 mW cm^−2^ [60]. Wang et al. [61] have developed a high-performance-based A-site cation-layered EuBa_0.5_Sr_0.5_Co_2-x_Fe_x_O_5+δ_ (EBSCF_0.4_-20RuO_2_) bifunctional perovskite that can be used as an efficient electrocatalyst for OER and water splitting.

## 5. Perovskite Electrocatalysts with A-Site Deficiency and Substitution

Most of the perovskite-based catalysts reported in the literature act as bifunctional catalysts, and they can be used for ORR and OER. Basically, perovskite-based catalysts exhibited superior catalytic activity for ORR and OER compared to the conventional noble metal catalysts such as Ru, Ir, Pt, and Co [62,63,64,65]. In the following section, the fabrication of perovskite catalysts with different variations for achieving the best catalytic response is briefly discussed.

**Figure 8:**
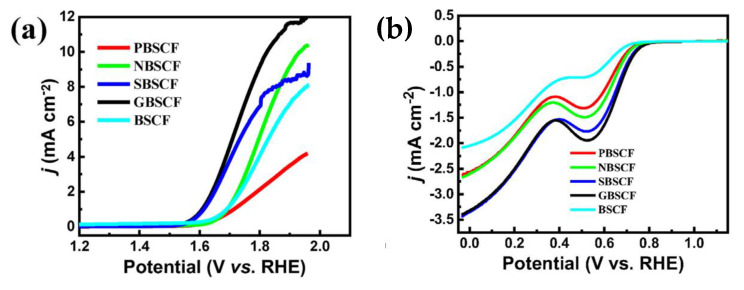
LSV of developed catalysts for (**a**) OER process and (**b**) ORR process at 1600 RPM in 0.1M KOH at a scan rate of 10 mV s^–1^. Copyright 2020 by the Elsevier Ltd. [66].

In general, the A-site cations in the perovskite catalyst are not directly involved in ORR or OER kinetics. Nevertheless, they facilitate the catalytic reaction indirectly. It is evident from many reports that the catalytic response has been significantly improved upon A-site substitution. For instance, double perovskite oxide materials LnBa_0.5_Sr_0.5_Co_1.5_Fe_0.5_O_6_ (LnBSCF, Ln=Pr, Nd, Sm, and Gd) were proposed as a bifunctional catalyst for ORR and OER [66]. It is considered that the double perovskite oxides are one of the most promising bifunctional electrocatalysts for ORR and OER due to their adjustable electronic structures for supporting the doping of different metal cations. A-site cation Ln can be replaced with other metal ions such asPr, Nd, Sm, and Gd. The developed catalysts investigated the ORR and OER activity in alkaline media.Among the catalysts, Gd-doped BSCF (GBSCF) exhibited superior catalytic performance for both OER and ORR in terms of catalytic current density as well as overpotential, as shown in Figure 8. Additionally noted is that the rest of the other metal-doped BSCF shows better activity compared to the undoped BSCF. This is due to the electronic defects and oxygen deficiencies generated by cation doping. The observed ORR catalytic currents follow the trend GBSCF > SBSCF > NBSCF > PBSCF > BSCF.

Further, very recently, Wei et al. developed the A-site deficient (Ba^2+^) perovskites from BaCo_0.4_Fe_0.4_Zr_0.1_Y_0.1_O_3−δ_(BCFZY) using nitrate–citrate complexing and the self-combustion method [67]. The results showed that a 10% A-site deficiency in BCFZY cathode dramatically reduced the area-specific resistance (ASR) from 0.73 Ω·cm^2^ to 0.13 Ω·cm^2^ at 650 °C in air. Additionally, A-site deficient BCFZY showed a power density of 730 mW·cm^−2^ at 800 °C when compared to the stoichiometric BCFZY. The EIS technique was employed to investigate the influence of A-site Ba^2+^ deficiency on the ORR catalytic activity of the BCFZY electrode in the temperature range between 650 to 800 °C. Among different stoichiometriesof Ba^2+^ investigated, the B_0.90_CFZY cathode exhibited the highest ORR catalytic activity. It was concluded that the A-site Ba^2+^ deficiency generated the oxygen vacancies in the lattice structure, which would accelerate the surface exchange and bulk diffusion of oxygen and subsequently improving the ORR catalytic activity. Overall, it is interesting that a proper deficiency in A-site cation could contribute to high effectiveness in promoting the ORR activity of the electrode. Another A-site deficient perovskite (La_0.8_Sr_0.2_)_1−x_MnO_3_ (x = 0, 0.02, 0.05) (LSM) and Fe-doped perovskite (La_0.8_Sr_0.2_)_0.95_Mn_0.5_Fe_0.5_O_3_ (LSMF) were developed by Leung and co-workers and used for catalytic ORR and OER applications [68].

The prepared perovskite catalysts showed significant ORR activity and found that onset potential at 0.5 mA cm^−2^ notably increases when A-site deficiency increases. For instance, the pristine (La_0.8_Sr_0.2_)_1-0_MnO_3_ (LSM1) displayed the onset potential of −0.15 V vs. Ag/AgCl and it was shifted to −0.14 V and −0.134 V for (La_0.8_Sr_0.2_)_1-0.02_MnO_3_ (LSM2), and (La_0.8_Sr_0.2_)_1-0.05_MnO_3_ (LSM3), respectively. This clearly indicates that the introduction of A-site deficiency into LSM-based catalysts can effectively enhance the ORR activity. Moreover, the ORR activity significantly increased, and onset potential shifted to 0.124 V vs. Ag/AgCl when Mn was substituted by Fe in LSM3. Further, the durability of the perovskite was examined, and it was found that LSMF exhibited the current retention of 97.1% after 10,000 s compared to the commercial 20 wt % Pt/C whose retention is 93.5% after 10,000 s. The developed A-site-deficient perovskites were also investigated for OER activity, and they showed the onset potential of 0.68, 0.60 and 0.57 V vs. Ag/AgCl for LSM1, LSM2 and LSM3, respectively. The onset potential further shifted to 0.53 V when Mn was substituted by Fe. The observed ORR and OER activity is mainly attributed to the valence state of oxygen species present on the surface of the perovskite catalysts and also the rate of O_2_^2−^/OH^−^ displacement and OH^−^ regeneration [69,70]. Therefore, the XPS technique was employed to evaluate the adsorbed oxygen species on the catalysis and revealed that LSMF has 49% when compared to the LSM3 (36%), which indicates amore adsorbed hydroxyl group and a correspondingly stronger covalence of Mn–O bond.

Manganite-based perovskites (LnMnO_3+*δ*_) were developed with different sizes of the A-site cation such as La, Pr, Sm, and Gd by using the glycine-nitrate process [71]. It has been realized that the perovskite lattice became more and more distorted upon lowering the size of the A-site cation. Meanwhile, the electrical conductivity markedly decreased. This effect is called a super exchange, and it is generated due to a lower overlap between the p-orbitals from the oxygen and the d-orbitals from the B-site cation. Finally, the fabricated perovskites were successfully used for ORR and nitric oxide reduction in the temperature range from 200 to 400 °C. The electrode materials showed a better catalytic response for nitric oxide reduction than ORR at a lower temperature, whereas both ORRand nitric oxide reduction activity were identical at a higher temperature. Among the different A-site cation incorporated perovskites, the activation energies for the ORR and nitric oxide reduction were similar for Sm and Gd-doped SmMnO_3+*δ*_and GdMnO_3+*δ*_ whereas they were slightly different for La and Pr-doped LaMnO_3+*δ*_and PrMnO_3+*δ*_. Overall, the Pr incorporated manganite displayed a low catalytic response due to the oxidation state of Pr(IV), which generates a higher concentration of cation vacancies in the perovskite structure. On the other hand, the catalytic activity of LaMnO_3+*δ*_ perovskite is very different from other perovskites due to the mobility of oxide ions at a higher temperature. Therefore, it was expected that the A-site cation would attract more electron density from oxygen when the size of the cation is decreased. Subsequently, the oxygen can attract more electron density from the B-site cation, making it easier to reduce the B-site cation.

## 6. Perovskite Electrocatalysts with B-Site Substitution

It is well established that the B-site cations are directly involved in the ORR and OER catalytic reaction [72,73]. Thus, B-site cations substitution is considered a very efficient way to improve the performance of the perovskite-based catalyst. The development of lanthanum-based double perovskite oxide (La_2__-x_Sr_x_NiMnO_6_ = LSNMO_-x_, x = 0, 0.2, 0.4, 0.6 and 0.8) bi-functional catalyst has been reported recently by Zhang and co-workers [74]. The study relates systematic doping of strontium (Sr) at B-site and its application in ORR and OER activities. The catalytic response orderly increased from 0.2 to 0.6% doping of Sr and then started to decrease at 0.8% due to the formation of NiO. It is well known that NiO has poor catalytic activity towards both OER and ORR. Further, ORR activity was investigated in 0.1 M KOH at 1600 rpm. The onset potential and half-wave potential of LSNMO-x samples systematically increase with increasing Sr doping (x ≤ 0.6), attained the onset and half-wave potential of 0.86 V and 0.72 V vs. RHE, respectively, for LSNMO_-0.6_.

**Figure 9:**
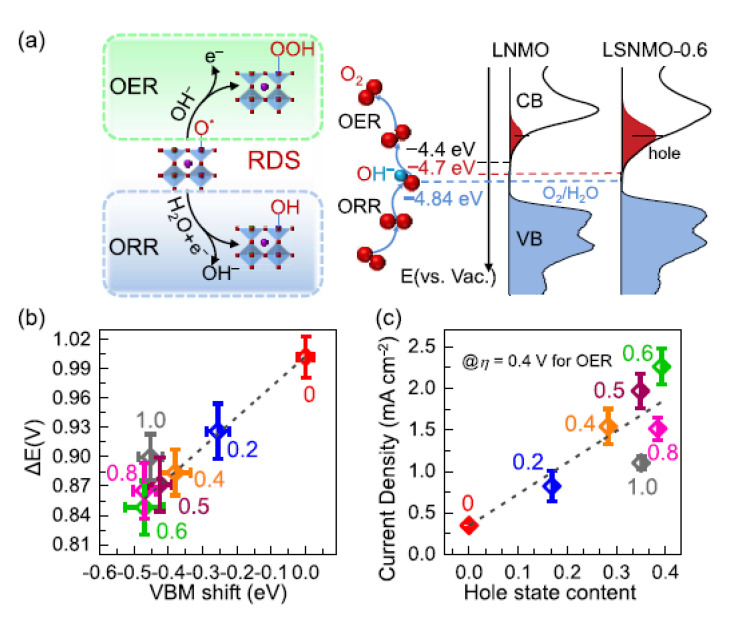
(**a**) Left panel: rate-determining step (RDS) of the OER (the formation of the O−O bond in the OOH^–^ adsorbate) and the ORR (OH^–^ regeneration on the transition metal site). Right panel: schematics diagram for the electronic structure near Ef at the vacuum level scale at pH = 14; the CBM level for LNMO is at −4.4 eV and the redox level for O_2_/H_2_O is located at −4.84 eV. (**b**) ΔE values as a function of VBM shift. (**c**) Current densities at an overpotential of 0.4 V for the OER as a function of hole state content. Copyright 2021 by the American Chemical Society [74].

In contrast, undoped LNMO showed the onset and half-wave potential of 0.78 V and 0.68 V, respectively, which were about 78 mV and 33 mV positive shifts compared to the LSNMO_-0.6_. Further, the electronic structure of the catalyst was analyzed by valence band X-ray photoelectron spectroscopy (VB XPS) and it was deduced that the valance band shifted towards Fermi level (Ef) with the O 2p band upon Sr doping. This electronic modulation influences the hybridization of O 2p with Ni/Mn 3d, which favours the electron exchange at the catalyst−intermediate interface. As depicted in Figure 9, the increase of occupied states near Ef induced the efficient orbital overlapping with absorbed oxygen species, thereby OH^−^ regeneration is facilitated by O_2_^2^^−^/OH^−^ exchange. In addition, this hybridization process also minimizes the charge–transfer gap between TM 3d and O 2p, which promotes the OER kinetics. Additionally, the significant change of hole states is attributed to the increasing concentration of high-valent Ni^3+^ introduced by Sr.

Zhou and co-workers proposed a bifunctional perovskite catalyst La_0.8_Sr_0.2_MnO_3_ (LSM) in the form of nanoparticles with the size of 30 to 80 nm for ORR and OER applications, and it was synthesized by the polymer-assisted chemical solution (PACS) method [75]. The catalyst was fabricated in different forms, such as A-site cation deficient (La_0.8_Sr_0.2_)_0.9_5MnO_3−δ_ (ALSM) and also the A-site cation deficient with the B-site cobalt-doped (La_0.8_Sr_0.2_)_1−x_Mn_1−x_Co_x_O_3−δ_ (x = 0.05 and 0.1 for LSMC5 and LSMC10, respectively). In terms of ORR activity, LSM displayed the onset potential at ca. −0.09 V vs. Ag/AgCl, which significantly low compared to the same material reported by another group [76]. It was expected that the enhanced ORR activity was attributed to the particle size of the LSM which promotes the oxygen transfer and diffusion between the nanoparticles to enable the catalysis at early onset potential [77]. Further, A-site-deficient ALSM showed poor ORR activity in terms of onset potential (−0.12 V) and half-wave potential (80 mV increase compared to LSM). The XRD data revealed that the crystallinity of 5% A-site deficiency ALSM was decreased notably, and this directly influences the ORR performance. Nevertheless, Co-doped catalysts (LSMC5 and LSMC10) have similar onset potential of LSM, but 15–20 mV reduced half-wave potentials due to increased Mn^4+^ content and consequently, which facilitates the orbital hybridization for stronger metal–oxygen bond formation and driving force for O^2−^/OH^−^ exchange [78].

As reported by other groups, LSM is a very poor catalyst for OER activity due to insufficient electrons in the structure and can hardly deprotonate oxyhydroxide groups and form peroxide ions [79]. However, the catalytic response of LSM was drastically improved when generating the A-site deficiency and oxygen vacancy. For instance, 170% in OER current density at 0.8 V enhanced for ALSM due to increasing the oxygen vacancy as it adsorbs highly oxidative oxygenated species and OH^−^ in alkaline media. Further, Co-doped LSMC5 and LSMC10 outshine ALSM due to increased hybridization of six-fold Co-O structure support for the formation of stronger O-2p, which boost the charge transfer between B-site cations and adsorbates.

**Figure 10:**
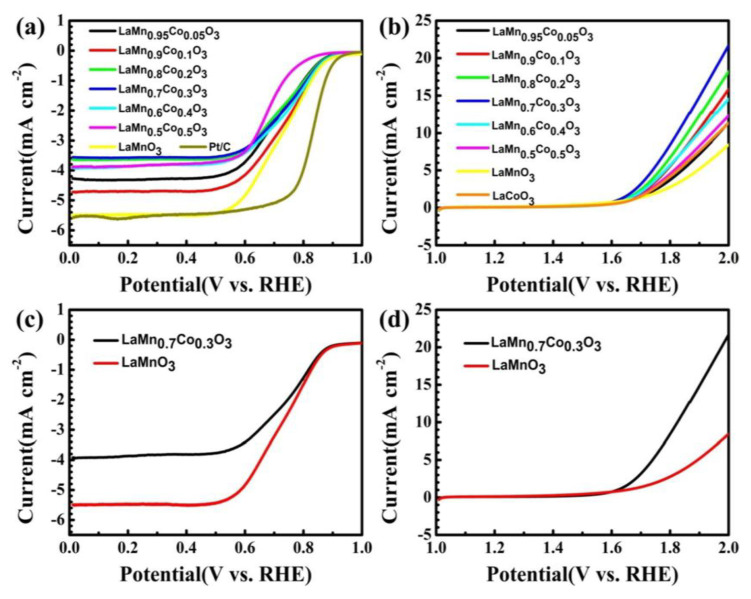
(**a**) The LSV curves of LaMn_1−x_Co_x_O_3_ and commercial 20 wt % Pt/C for the ORR at 1600 rpm. (**b**) The LSV curves of LaMn_1−x_Co_x_O_3_ and LaCoO_3_ for the OER at 1600 rpm. The LSV curves of LaMn_0.7_Co_0.3_O_3_ and LaMnO_3_ for the ORR (**c**) and OER (**d**) at 1600 rpm. Copyright 2018 by the Wiley-VCH Verlag, GmbH & Co. KGa, Weinheim [80].

Xiao et al. developed rare-earth metals lanthanum manganate (LaMnO_3_)-based bifunctional perovskite-type oxide catalyst for ORR and OER activities [80]. The fabricated materials showed an excellent catalytic response towards the ORR performance but a very poor catalytic response towards OER. To resolve this issue, a sol–gel method followed by a calcination process was employed to substitute the cobalt into the LaMnO_3_. Citric acid was used as a complexing agent. The sol changed intoa gel after evaporation of the solvent. Subsequently, the calcination process was performed to remove the excess citric acid and degrade the nitrate ion to produce the perovskite oxides LaMn_1−x_Co_x_O_3_. This calcination process created porosity in the material due to the elimination of citric acid and the thermal decomposition of nitrate. The surface area and pore structure of the perovskite LaMn_0.7_Co_0.3_O_3_ were investigated and it was found that the specific surface area is about 15.20 m^2^g^−1^, and the pore sizes ranged between 8 to 30 nm and suggesting that they are associated with the mesoporous material. The catalytic response of LaMn_1−x_Co_x_O_3_ towards ORR and OER isshown in Figure 10. As mentioned above, the developed materials exhibited an excellent catalytic response for ORR. In particular, among the different ratios of Mn/Co tested, the LaMn_0.5_Co_0.5_O_3_showed 130 mV more negative onset potential than the commercial Pt/C catalyst towards ORR activity. On the other hand, LaMn_0.7_Co_0.3_O_3_ (1.82 V vs. RHE) has superior catalytic performance towards OER among the series of LaMn_1−x_Co_x_O_3_ (x = 0, 0.05, 0.1, 0.2, 0.3, 0.4, and 0.5) (1.82 V to 1.97 V).

It is expected that the poor OER catalytic response of Co-free LaMnO_3_ is associated with the average Mn–Mn bond distance, which is considered too long to form the O–O bond [81]. This is because the formation of O–O bond plays a very imperative role in the OER process. Therefore, the O–O bond formation process can be adjusted by the doping of Co into the perovskite LaMnO_3_. This doping process effectively shortens the lattice space, and consequently, Mn–Mn distance becomes reduced. In addition, the doping of Co also induced the O–O bond formation on the surface of LaMnO_3_.

Although the Co-based materials have shown excellent electrochemical performance for the ORR activity, they have some disadvantages when used as solid oxide fuel cell (SOFC) cathode due to thermo-mechanical mismatch with the yttria-stabilized zirconia (YSZ) electrolyte and chemical side reaction [82]. Therefore, a buffer layer could always be employed to avoid the formation of insulating secondary phases between YSZ and Co-based cathode [83]. In this regard, a Co-free perovskite oxide material was developed by using the mixture of Pr, Ba, Sr, and Fe cations in the presence of routinely used complexing agents such as citric acid and polyethylene glycol [84]. Two types of perovskite oxides, Pr_0.5_Ba_0.5_FeO_3-*δ*_(PBF) and Pr_0.5_Ba_0.4_Sr_0.1_FeO_3-*δ*_(PBSF), were prepared by the Pechini method. The electrochemical impedance spectroscopy (EIS) technique was performed to evaluate the electrocatalytic activity towards ORRin YSZ electrolyte-supported symmetric half-cells in the presence of air. It was found that PBF exhibited the area-specific resistance (ASR) value of 0.07 Ω·cm^2^ compared to the value obtained for Sr-doped PBSF (0.05 Ω·cm^2^) as a result of enhanced electrical conductivity.

Perovskite-based bifunctional catalysts were fabricated by using alkaline earth metals (Ba, Sr, Ca, and Mg)-doped bismuth iron oxides Bi_0.6_M_0.4_FeO_3_ (BFO) [85]. These metal-doped particles were mixed with carbon black to improve the electronic conductivity. Among these metals, the catalyst prepared with Ca (Bi_0.6_Ca_0.4_FeO_3_ (BCFO)) exhibited remarkable OER and ORR catalytic activity than the pristine BiFeO_3_ (BFO) in alkaline media at room temperature.For instance, BFO showed an onset potential of 0.633 V vs. RHE for ORR activity, and it was shifted to 0.705 V upon doping with Ca (BCFO). In addition, the current density increased from 3.23 × 10^−9^ (pristine BFO) to 5.17 × 10^−8^ mA cm^−2^ (BCFO). OER activity was also examined for pristine BFO and also alkaline earth metal-doped BFOs. Again, BCFO showed higher catalytic performance over other BFOs. As reported, BCFO exhibited the current density of 6.93 mA cm^−2^ (*j*_OER_), at a fixed overpotential of 0.42 V (1.65 V vs. RHE) for OER, which is approximately two folds higher value compared to the other catalysts (3.06 to 4.04 mA cm^−2^).

## 7. Perovskite Electrocatalysts with A-Site and B-Site Co-Substitution

The co-doping at the A-site and B-site is afascinating method for enhancing the electrocatalytic performance of perovskite material towards ORR and OER applications. In this regard, a significant number of works have appeared in the literature. For instance, calcium and indium co-doped at A and B-sites of BaFeO_3−δ_ respectively was proposed for ORR application [86]. Although the doping of high valence cation can improve the structural stability of the catalyst but diminish the oxygen vacancy concentration, it affects the overall catalytic performance. Therefore, to obtain a stable catalyst, light isovalent and lower-valence elements such as 5% Ca^2+^ are substituted in the Ba^2+^ site and 5% In^3+^ substituted in the Fe^3+^/Fe^4+^ site. The catalytic performance of the co-doped catalyst was investigated by the EIS technique. The co-doped Ba_0.95_Ca_0.05_Fe_0.95_In_0.05_O_3−δ_ (BCFI) exhibited a low polarization resistance of 0.038 Ω·cm^2^ compared tothe calcium (0.127 Ω·cm^2^)and indium (0.526 Ω·cm^2^) individually doped catalyst. Additionally, BCFI displayed a higher catalytic response for ORR among them due to the stabilization of the catalyst cubic structure, improvement of conductivity as well as oxygen vacancies and enhancement of oxygen transport rate. In another study, calcium co-doped in A and A’-site of PrBaCo_2_O_5+δ_ catalyst was developed for ORR activity [87]. It was found that calcium ion substitution at the Ba site significantly enhanced the electrical conductivity of the catalyst. Substitution of low ionic radii of Ca^2+^ (1.120 Å) into PrBaCo_2_O_5+δ_can decrease the mismatch between the ionic radii in A site of Pr ^3+/4+^ (1.126 Å) and A’ site of Ba^2+^ (1.420 Å) and can reduce unit-cell volume, and it supports to reduce the segregation and to improve structural stability and oxygen-ion mobility.

Qiang et al. developed nanoparticle-based La_1__-y_Sr_y_Ni_1−x_Fe_x_O_3_ catalyst (x = 0, 0.1, 0.3, 0.5, 0.7, 1; y = 0.2, 0.4, 0.6) by co-doping Fe and Sr into the LaNiO_3_ for OER applications [88]. TEM image confirmed that the size of the catalyst was 25 nm. The fabricated catalyst was applied for the OER activity and the electrochemical performance was compared with LaNiO_3_ and LaFeO_3_ and also commercial RuO_2_. LaNi_0.5_Fe_0.5_O_3_ displayed an overpotential of 340 mV vs. RHE than the undoped LaNiO_3_ (473 mV). Further, the overpotential decreased to 320 mV for La_0.4_Sr_0.6_Ni_0.5_Fe_0.5_O_3_ upon co-doping of Fe and Sr, which is almost the same overpotential as the commercial RuO_2_ catalyst. These results clearly indicate that Fe doping at B-site is the prime cause for reducing the overpotential, and also, the Sr doping in the A-site enhances the catalytic performance. Further, the durability of the catalyst was investigated, and it was found as 9.3% activity loss after 10 h.

## 8. Perovskite Electrocatalysts with Oxygen Vacancies

**Figure 11:**
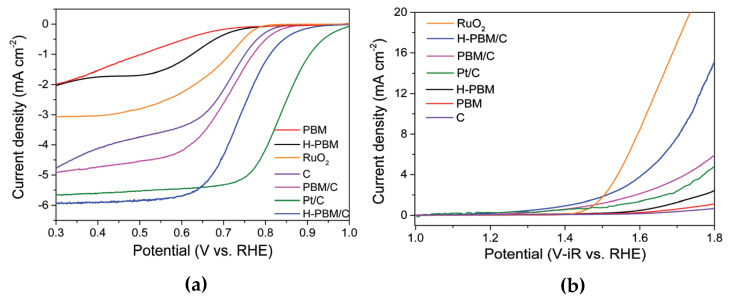
(**a**) ORR (**b**) OER polarization curves of PBM, H-PBM, RuO_2_, C, PBM/C, Pt/C, and H-PBM/C in O_2_-saturated alkaline solution (rotating speed: 2000 rpm; scan rate: 5 mV s^−1^; electrolyte: 0.1 M KOH). Copyright 2016 by the Royal Society of Chemistry [89].

Generating the oxygen vacancies in the catalyst is also a promising strategy to develop an efficient catalyst that promotes fast oxygen ion diffusion rates for ORR and OER catalytic reactions.A simple annealing method was used to fabricate a layered PrBaMn_2_O_5+δ_ (H-PBM) from its pristine form of Pr_0.5_Ba_0.5_MnO_3−δ_ (PBM) [89]. The carbon was incorporated to improve the conductivity of the catalysts in the perovskite oxide to carbonratio of 1:3. Figure 11 displays the ORR activity of the developed catalysts and catalytic response compared with commercial Pt/C and RuO_2_. In the absence of carbon, both PBM and H-PBM showed a very low catalytic response.

On the other hand, the catalytic activity was drastically improved in terms of onset potential and catalytic limiting current densities when carbon was introduced into the catalysts. The onset potential appeared at 0.68 and 0.74 V vs. RHE for PBM/C and H-PBM/C (Figure 11A). As it can be seen from Figure 11B, H-PBM/C exhibited a higher current density than the commercial Pt/C. Similarly, a higher catalytic response with the lowest onset potential for OER activity was achieved in H-PBM/C compared to the other catalysts. However, the commercial RuO_2_ displayed a higher current density. These results demonstrate that the conversion of PBM into layered H-PBM effectively enhances both ORR and OER activities. The higher catalytic activity of H-PBM is attributed to different factors such as (i) structural evolution from cubic to layered structure to support the O–O bonds formation, (ii) e_g_ filling of the transition metal which determine the binding strength of intermediate and (iii) introduction of additional oxygen vacancies as confirmed from δ values 0.13 and 0.63 for PBM and H-PBM. The oxygen vacancy was achieved by the reduction of the transition metal ions by H_2_ treatment.

María et al. investigated the structural effects and oxygen quantity of LaNiO_3_ on the ORR activity [90]. The catalyst was prepared at different temperature ranges from 500 to 1000 °C and catalytic performances were tested. The catalyst prepared at 500 °C did not show any catalytic response and revealed that the perovskite structure formed at a lower temperature. Among the temperatures, the catalyst at800 °C exhibited a higher current density. It has been reported that the observed higher catalytic response is associated with the generation of oxygen vacancies in LaNiO_3,_ and it was achieved by the reduction of B-site cation Ni^3+^ into Ni^2+^. Moreover, the decline of catalytic response at higher temperature (>900 °C) owes to the formation of large-size LaNiO_3_ particles with lower surface area.

An in-situ reducing and phosphating process was utilized to fabricate the nanoparticle (~20 nm)-based perovskite oxides/(CoFe)P_2_ heterointerfaces on the surface of La_0.8_Sr_1.2_Co_0.2_Fe_0.8_O_4+δ_ (LSCF) layered perovskite oxides and was successfully applied for OER applications [91]. Firstly, LSFC oxides were reduced (r-LSFC) in Ar/H2 atmosphere. During this process, Co and Fe ions can be exsolved to form Co-Fe alloy nanoparticles and adsorbed on the surface of the parent perovskite oxides. Then, the subsequent phosphating process at low temperatures induces the formation of island-like (CoFe)P_2_ nanoparticles (LSCF-P). The developed catalysts were tested for OER activity and compared with the commercial IrO_2_.The observed onset potential for OER is 1.55 V, 1.52 V, 1.50 V and 1.48 V for the r-LSCF, LSCF, r-LSCF-P and IrO_2_, respectively. The enhanced catalytic response of (CoFe)P_2_/LSCFis due to the improved conductivity and also the charge–transfer process.

## 9. Perovskite Electrocatalysts with High Surface Area

In general, an extremely hightemperature is used in the calcination process for the preparation of perovskite catalysts. In this process, perovskite catalysts are obtained in bulk form with a very low surface area. It affects the catalyst performance in several ways, including (i) utilization of the active sites, (ii) limit the wetting of the electrode by the electrolyte, and (iii) constrained ion transportation and electron transfer. Therefore, to obtain a higher surface area, many approaches have been explored in the literature.

To achieve a higher surface area of the catalyst, Jani and co-workers have employed the development of a nanotube form of perovskite oxide Ba_0.5_Sr_0.5_Co_0.8_Fe_0.2_O_3−δ_ (BSCF) by using one of the most promising platforms, namely nanoporous anodic aluminium oxide (NAAO) [92]. The combination of 10 wt % BSCF nanotubes with BSCF nano spherical powder showed the specific surface area (15.02 m^2^ g^−1^) and porosity (27%)compared to the pristine BSCF (surface area = 9.28 m^2^ g^−1^ and porosity (23%). The porosity increased to 35% upon the addition of 20 wt % BSCF nanotubes. The resulting BSCF_10NTs:90NPs_,demonstrated high ORR performance with the best area-specific resistance (ASR) value of 0.06 Ω·cm^2^ at 800 °C, which was 50% enhancement relative to the pristine BSCF.

Another approach, namely laser ablation synthesis in solution (LASiS) technique, was used to fabricate the perovskite-based LaMnO_3+d_ nanoparticles (LMO NPs) with a higher surface area and successfully used for ORR activity [93]. The bulk LMO in size above 200 nm was observed as a result of the long duration of the solid-state reaction. Nevertheless, the average LMO particle size drastically reduced down to 12 nm after the LASiS process. The synthesized LMO NPs coated on the GC electrode and used for catalytic ORR in 0.1 M KOH.Meanwhile, the commercial Pt/C (20 wt % Pt) was also tested under the same conditions for comparison. The bulk LMO exhibited a very low catalytic response for ORR, owing to a low surface area/large particle size (>200 nm). Similarly, LASiS-treated LMO also displayed a poor catalytic activity whichis due to its amorphous nature. In contrast, 600 °C-annealed LMO (LASiS LMO 600) and 800 °C-annealed LMO (LASiS LMO 800) catalysts showed enhanced catalytic current at 0.9 V vs. RHE, which indicates that the combination of increased surface area, reduced particle size (<55 nm) and crystalline quality dramatically amplified the catalytic response. Additionally, the observed catalytic current at 0.9 V was almost identical to the commercial Pt/C catalyst. Overall, the LASiS LMO 800 catalyst showed an ORR mass activity of 22 µA/µg_oxide_, which was nearly 20 times higher than that of the bulk LMO (1.09 µA/µg_oxide_).

**Figure 12:**
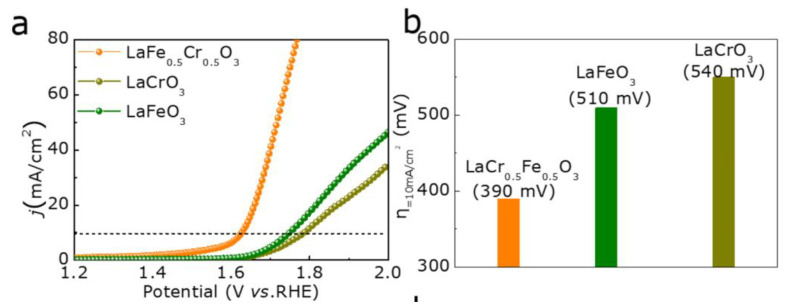
(**a**) The polarization curves and (**b**) overpotential at 10 mA/cm^2^ of LaFeO_3_, LaCrO_3_ and LaCr_0.5_Fe_0.5_O_3_ for OER. Copyright 2020 by the Springer Nature [94].

The conventional sol–gel used to develop the perovskite-type LaCr_x_Fe_1−x_O_3_ nanoparticle with a higher surface area for OER applications [94]. As shown in Figure 12, the linear sweep voltammetry (LSV) of LaCr_0.5_Fe_0.5_O_3_ reveals an OER overpotential of 390 mV at 10 mA/cm^2^, which is significantly lower than 510 mV of LaFeO_3_ and 550 mV of LaCrO_3_. Further, the R_CT_ value evaluated for these materials and found that LaCr_0.5_Fe_0.5_O_3_ exhibited a lower value compared to their counterparts LaFeO_3_ and LaCrO_3_, demonstrating that LaCr_0.5_Fe_0.5_O_3_ owns the fastest OER rates and the optimal charge transfer capability. The observed higher catalytic OER activity of LaCr_0.5_Fe_0.5_O_3_ is associated with the stronger bond between the metal atom and the adsorbed oxygen-containing substance, along with the greater electrical conductivity.

## 10. Nanomaterials Incorporated Perovskites Catalysts

Although the pristine form of perovskite catalysts showed excellent activity, they have inherent drawbacks such as low surface area and high resistivity, which severely affect the electrochemical performances. The integration of nanomaterials with perovskite oxides is an effective way to improve the performance of the perovskite catalysts as the conductivity enormously improved. To date, several nanomaterials have been incorporated with perovskite oxides and successfully demonstrated for oxygen electrode reactions.

**Figure 13:**
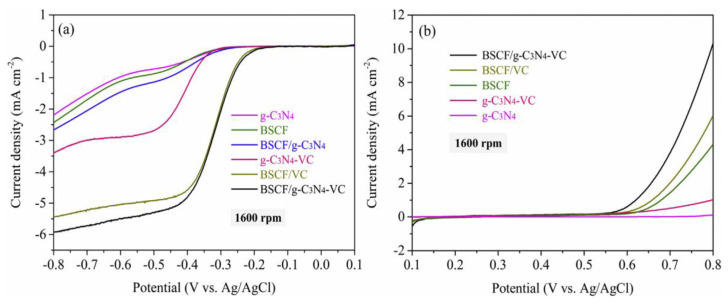
(**a**) ORR catalytic activities of g–C_3_N_4_, BSCF, BSCF/g–C_3_N_4_, g–C_3_N_4_–VC, BSCF/VC and BSCF/g–C_3_N_4_–VC (**b**) and OER catalytic activities of g–C_3_N_4_, BSCF, g–C_3_N_4_–VC, BSCF/VC and BSCF/g–C_3_N_4_–VC measured by LSV at a rotating rate of 1600 rpm in O_2_-saturated 0.1 M KOH solution at a scan rate of 5 mV s^−1^. Copyright 2020 by the Elsevier [12].

Dengjie and co-workers developed a bifunctional perovskite-based composite material for ORR and OER applications [12]. The composite material was fabricated by the combination of Ba_0.5_Sr_0.5_Co_0.8_Fe_0.2_O_3−δ_ (BSCF), graphitic carbon nitride (g-C_3_N_4_) and Vulcan carbon (VC). Although g-C_3_N_4_ has several advantages such as high nitrogen content and good chemical stability, it has inherent drawbacks: inferior electronic conductivity and low surface area. Figure 13 represents the electrocatalytic activity towards ORR and OER activity at various electrodes recorded at a rotating rate of 1600 rpm in O_2_-saturated 0.1 M KOH solution at a scan rate of 5 mV s^−1^. The composite BSCF/g-C_3_N_4_-VC showed a drastically enhanced catalytic response than the individual or other combination of materials due to the improved conductance by the VC. As shown in Figure 13a, g-C_3_N_4_ and BSCF displayed the onset potential of −0.28 V, whereas BSCF/VC has an onset potential of −0.16 V. Further, it is noted that no significant change in the onset potential between pure g–C_3_N_4_ and g–C_3_N_4_–VC, which indicates that there is a synergistic effect between BSCF and VC. Additionally, the catalytic response is amplified in magnitude after the addition of BSCF into g–C_3_N_4_–VC, which suggests that perovskite BSCF plays a critical role in improving the catalytic activity of the composite. Additionally, it was proposed that the sheet-like structure and the negative zeta potential of g-C_3_N_4_ induced the diffusion of electrolyte all over the active sites through effective dispersing of BSCF and VC in the BSCF/g–C_3_N_4_–VC composite.

**Figure 14:**
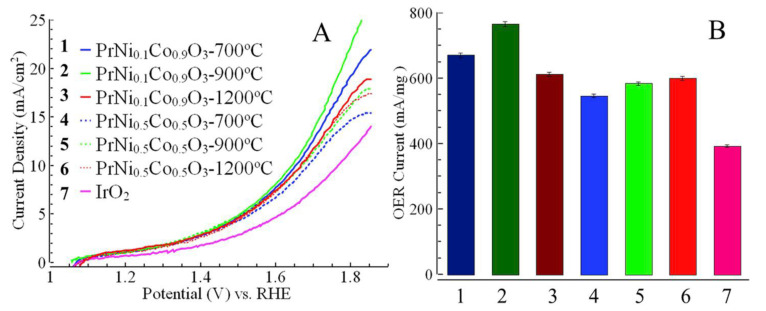
OER performance of PrNi_x_Co_1−x_O_3_-graphene composite in terms of (**A**) polarization current densities and (**B**) mass activities in comparison to state-of-the-art IrO_2_ catalyst. Copyright 2020 by the Elsevier [65].

Very recently, Alevtina and co-workers demonstrated graphene-supported praseodymium (PrNi_x_Co_1−x_O_3−δ_) and samarium (SmNi_x_Co_1−x_O_3−δ_) perovskites for ORR and OER catalytic applications in alkaline media [65]. The glycine-nitrate-based sol–gel method was used to synthesis these perovskites. The particle size varied depending upon the temperature and the size of 30, 35 and 40 nm obtained at 700, 900 and 1200 °C, respectively, for PrNi_x_Co_1−x_O_3−δ_. The rationale behind that is, at a higher temperature, the number of oxygen vacancies increases and therefore the oxidation states of the B-site metal are decreased in order to maintain overall electroneutrality. This leads to crystal lattice expansion. In terms of catalytic activity, the pristine form of perovskite displayed a high overpotential and poor ORR activity. However, while incorporating the graphene into the perovskite, the composites gradually start to enable the catalysis at lower overpotentials with higher current densities. It was found that Sm-based catalysts demonstrated overall higher ORR activity compared to the Pr-based catalysts. Further, OER activity was investigated with the PrNi_x_Co_1−x_O_3_-graphene composite and is depicted in Figure 14. The highest catalytic response was achieved for the perovskite prepared with the nickel content ofx = 0.1 and 700 °C. Overall, it showed 60 mA/mg for ORR and 680 mA/mg for OER, which is nearly 50% higher activity than the conventional IrO_2_ catalyst.

In another report, the facile and low-cost graphene oxide (GO) and carbon Vulcan (C)-integrated bimetallic Fe-Mn oxide perovskite-type nanoparticles were synthesized and utilized for ORR activity in neutral media [95]. Based on XPS, the ABO_3_ perovskite structure was stabilized with high oxidation states of iron and manganese, which are beneficial for ORR. The bare FeMnO_3_ exhibited the catalytic response for ORR at −0.6 V vs. SCE in addition to a couple of redox peaks between 0.4 to 0.6 V, owing to the redox reaction of manganese and iron species on the electrode surface. However, the catalytic peak at −0.6 V was shifted to −0.3 V upon integration of the graphene oxide or carbon Vulcan into the FeMnO_3_. Further, the EIStechnique was used to evaluate the conductivity of the nanomaterials and found that C-FeMnO_3_ and GO-FeMnO_3_ showed the double layer capacitance (C_DL_) of 2.36 mF cm^−2^ and 0.02 mF cm^−2^,respectively. The obtained significantly higher C_DL_ value indicates that the surface area for oxygen ion adsorption at the interface of the C-FeMnO_3_ electrode is higher than that of GO-FeMnO_3_. As presumed, the aggregation and restacking process during synthesis of GO sheet cause the loss of effective, accessible surface area and also these process might hinder the dispersion of metal oxides particles which lower metal oxide utilization efficiency [96]. As a result, GO-FeMnO_3_ exhibited a higher charge transfer resistance (R_CT_) value (429 Ucm^−2^) than the C-FeMnO_3_ (69.8 Ucm^−2^). Overall, these superior electrochemical characteristics of C-FeMnO_3_ lead to a faster and better response for ORR compared to that of GO-FeMnO_3_.

Further, the nitrogen-doped reduced graphene oxide (r-GO)-incorporated perovskite-type bifunctional hybrid catalysts were developed for ORR and OER activity [97]. The glycine-nitrate-based combustion process was carried out for the synthesis of double perovskite bi-functional hybrid catalysts such as Ba_0.5_Sr_0.5_Co_0.8_Fe_0.2_-O_3−δ_ (BSCF) and LnBa_0.5_Sr_0.5_Co_1.5_Fe_0.5_O_5+δ_ (LBSCF, Ln = Nd, Sm, and Gd). The nitrogen doping was executed by the hydrothermal reaction. In term of OER activity, these perovskite materials have the activity in the order of NBSCF (0.361 V) < SBSCF (0.372 V) < GBSCF (0.390 V) < BSCF (0.416 V) at 10 mA cm^−2^. It is clear that the NBSCF catalyst exhibited a lower onset potential than the other perovskite-based catalysts studied. Additionally, the catalytic response was compared with the commercial carbon-supported Ir nanoparticles (Ir/C) and found that the activity of NBSCF almost identical.

Moreover, these perovskite catalysts also demonstrated significant ORR activities in 0.1 M KOH at room temperature. The observed on-set potential of BSCF, GBSCF, SBSCF, and NBSCF are 0.790, 0.805, 0.797, and 0.791 V vs.RHE, respectively at −0.1 mA cm^−2^. Although NBSCF exhibited the best performance for the ORR among the perovskite-based materials, a significant performance gap is still observed between the Pt/C and NBSCF catalyst. The NBSCF catalyst was −230 mV less active than Pt/C at the half-wave potential for the ORR. Hence, the nitrogen-doped r-GO was incorporated with the perovskite to improve the catalytic activity. The onset potential of NBSCF improved from 0.791 V to 0.988 V when integrated the N-rGO with NBSCF (NBSCF/N-rGO), which was almost similar to the value obtained with the commercial Pt/C catalyst (0.990 V).

Further, based on the Koutecky–Levich analysis, the electron transfer number of NBSCF/N-rGOwas estimated to be 4.0 for the ORR, indicating that the reaction occurred mainly through a four-electron pathway. The observed excellent bi-functional performance of NBSCF/N-rGO can be associated with the high surface area with a microstructure. The surface area of NBSCF significantly increased from 4.6 to 119.6 m^2^ g^−1^ upon the incorporation of N-doped rGO. Additionally, the electrical conductivity of NBSCF/N-rGO enormously increased, as evidenced by the decreasing R_ct_ value of NBSCF from 0.568 to 0.029 Ω·cm^2^.

Carbon nanofiber-embedded Ba_0.5_Sr_0.5_Co_0.8_Fe_0.2_O_3−δ_ (BSCF@C) electrode material fabricated by sol–gel method for ORR and OER [98]. The catalytic activity of BSCF@C was investigated in 1 M LiTFSI/DMSO electrolyte in the potential window of 2 to 4.5 V vs. Li^+^/Li. A well-established cathodic peak appeared at 2.5 V attributed to O_2_^−^ generation during the discharge process. In addition, two anodic peaks were apparent at 3.2 V and 3.8 V, which are associated with the multiple Li_2_O_2_ oxidation pathways during the charging process. The observed catalytic response of BSCF@C was compared with BSCF and no significant change was found with ORR activity but there was a remarkably increased OER activity. Further, the stability of the catalytic response was investigated by continuous cycling in the operating window of 2 to 4.5 V. The catalytic response of BSCF significantly decreased after 250 cycles. In contrast, the catalytic activity of BSCF@C nanofibers was still considerable after 250 cycles. These results revealed that BSCF@C could not only facilitate the ORR and OER catalysis but also hinder BSCF from aggregation during the discharge–charge cycling process.

**Figure 15:**
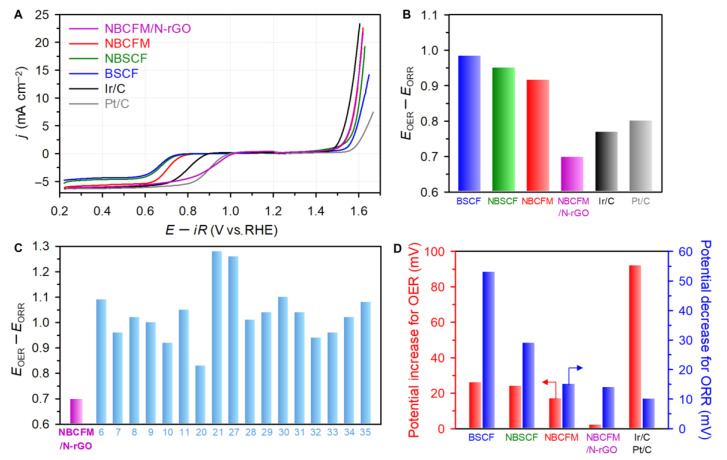
Bifunctional oxygen electrode activities and durabilities of the perovskites, NBCFM/N-rGO, and precious metal catalysts. (**A**) OER/ORR polarization curves for perovskite-based catalysts and precious metal catalysts. (**B**) Oxygen electrode activities (E_OER_−E_ORR_) of perovskite-based catalysts and precious metal catalysts. (**C**) Comparisons of oxygen electrode activities of NBCFM/N-rGO with the previously reported bifunctional catalysts. The numbers on the *x*-axis denote reference numbers. (**D**) OER potential increase and ORR potential decrease of perovskite-based catalysts and precious metal catalysts after durability tests. Copyright by the American Association for the advancement of Science [99].

Kim et al. have developed a bifunctional catalyst for ORR and OER-based on triple perovskite, Nd_1.5_Ba_1.5_CoFeMnO_9−d_ (NBCFM) [99]. The catalytic activity and durability of NBCFM were compared with single Ba_0.5_Sr_0.5_Co_0.8_Fe_0.2_O_3−δ_ (BSCF) and double perovskites [NdBa_0.5_Sr_0.5_Co_1.5_Fe_0.5_O_5+δ_ (NBSCF)]. The size of perovskites was investigated by the Scherrer equation and was estimated to be 5.84, 4.82, and 4.83 nm for BSCF, NBSCF, and NBCFM, respectively. Additionally, the crystal structure data revealed that BSCF, NBSCF, and NBCFM are present as cubic, orthorhombic, and tetragonal structures, respectively. As depicted in Figure 15, the ORR and OER catalytic activities of perovskites were investigated in 0.1 M KOH and compared with commercial Ir/C and Pt/C catalysts. It is clear that the triple perovskite (NBCFM) catalyst exhibits superior OER activity with higher current density and lower overpotential when compared to the other two catalysts.

The observed overpotential is 359 mV for NBCFM, 374 mV for NBSCF and 395 mV vs. RHE for BSCF at the current density of 10 mA cm^−2^. Similarly, NBCFM exhibits the best ORR activity among the three perovskite catalysts analysed. The observed half-wave potential for the ORR activity is 0.698 V, 0.653 V and 0.641 V for NBCFM, NBSCF and BSCF, respectively. Based on the Koutecky–Levich analysis, the electron transfer number was estimated as 3.9 for NBCFM, which was significantly higher than the other two catalysts, such as 3.6 for NBSCF and 2.8 for BSCF. Again, NBCFM showed a higher diffusion-limited current density of −5.9 mA cm^−2^, which is notably higher than those of NBSCF (−5.1 mA cm^−2^) and BSCF (−4.8 mA cm^−2^).

Among the catalyst, the NBCFM exhibited excellent oxygen electrode activity. However, the activity is relatively lower compared to the commercial electrode due to the low electrical conductivity of fabricated perovskites. To overcome this limitation, perovskites were integrated with the nitrogen-doped reduced graphene oxide (N-rGO). No significant improvement was achieved towards the OER activity. However, NBCFM/N-rGO showed a half-wave potential of 0.889 V, which was 0.191 V higher than NBCFM and surpassed that of Pt/C (0.801 V). These excellent characteristics were associated with the combination of multiple factors, including enriched oxygen defects, low R_ct_, and small hybridization strength between O 2p and Co 3d orbitals.

## 11. Conclusions and Challenges

In summary, we overviewed the recent development and novel integration of advanced BFPECs, various synthesis routes, different sizes, morphological analyses and efficient electrocatalytic applications in ORR/OER behavior. BFPECs are found as promising biocatalysts that could be applied in the field of ORR/OER. Despite this, the electrode materials have broad electrocatalytic properties like high-surface to volume ratio, low-cost and high-performance energy storage device applications. This review mainly focused on bi-functional-based electrode catalysts with doping and co-doping of both A-site and B-site cation, generation of oxygen vacancies, and incorporation of nanomaterials, which shows exceptional ORR/OER activities under alkaline conditions. The mechanism associated with the stronger bond between the metal atom and oxygen-containing substrates is discussed elaborately along with their electrical conductivity. The few methods to synthesize the BFPECs are found to be tedious and very expensive. Consequently, current reviews onbifunctional supported perovskite catalystsare summarized, including nano particle size, electronic structure optimization, charge–transfer properties and electrocatalytic performance for both ORR and OER, which is a big challenge for current and future researchers.

## Data Availability

Data are contained within the article or supplementary material.

## Supplementary Materials

The following are available online at https://www.mdpi.com/article/10.3390/ma14112976/s1, Table S1: Summary of ORR and OER electrocatalytic activities of the reported various types of bifuntional based perovskite electrodecatalysts.
