# Supplementary material for: High-Efficiency of Bi-Functional-Based Perovskite Nanocomposite for Oxygen Evolution and Oxygen Reduction Reaction: An Overview
Source: Materials (Basel). 2021 May 31;14(11):2976. doi: 10.3390/ma14112976 (PMC8198805; doi:10.3390/ma14112976)
Supplement: Supplementary file 1 [file materials-14-02976-s001.zip › materials-1225092-supplementary.pdf]

Table S1. Summary of ORR and OER electrocatalytic activities of the reported various types of bifuntional based perovskite electrodecatalysts.

Methods	Electrocatalysts	Typical Morphology	Onset Potential ORR (V)	Onset Potential OER (V)	Electro-lytes (M)	Ref.
Oxalate precursor route	$CaCu_3Ti_4O_{12}$	Nanoparticles	0.83	1.50	0.1 KOH	[1]
Hot plate	Fluorinated-copper manganese oxide (FCMO)	-	0.94	1.62	0.1 KOH	[2]
Sol-gel	$La_{0.8}Sr_{0.2}Mn_{0.95}Sc_{0.025}O_{3-\delta}$	Micro structured particles	0.96	-	0.1 KOH	[3]
Pyrolysis	N-MWCNTs-Co	Foam like structure	0.334	0.364	0.1 KOH	[4]
Electro spinning	$LaNi_{0.85}Mg_{0.15}O_3$ (LNMO NFs)	Nanofiber	0.45	-	0.1 KOH	[5]
Polymer-assisted	$LaCo_{1-x}NixO_3$	Nanoparticles	-0.47	-	0.1 KOH	[6]
Wet-chemical	Ni & Mn promoted Co_3O_4	Mesoporous	0.87	-	0.1 KOH	[7]
Reverse hydrolysis	Nanostructured $LaNiO_3$/Nitrogen doped carbon (NC)	Nanoparticles	-	0.4	0.1 KOH	[8]
Sol-gel	$LaNi_{1-x}FexO_3$ (x= 0, 0.1, 0.2, 0.6)	Nanoparticles	-	0.64	0.1 KOH	[9]
Polymer-assisted chemical solution (PACS)	$La_{0.8}Sr_{0.2}MnO_3$ (LSM)	Nanoparticles	-0.09	-	0.1 KOH	[10]

References

1. Kushwaha, H.S.; Halder, A.; Thomas, P.; Vaish, R. $CaCu_3Ti_4O_{12}$; A bi-functional perovskite electrocatalyst for oxygen evolution and reduction reaction in alkaline medium. *Electrochim. Acta* **2017**, *252*, 532–540.
2. Rai, V.; Lee, K.P.; Safanama, D.; Adams, S.; Blackwood, D.J. Oxygen reduction and evolution reaction (ORR and OER) bifunctional electrocatalyst operating in a wide pH range for cathodic applications in Li-Air batteries. *ACS Appl. Energy Mater.* **2020**, *3*, 9417–9427.
3. Xu, M.; Sun, H.; Wang, W.; Shen, Y.; Zhou, W.; Wang, J.; Chen, Z.G.; Shao, Z. Scandium and phosphorous co-doped perovskite oxide as high-performance electrocatalysts for the oxygen reduction reaction in alkaline solution. *J. Mater. Sci. Technol.* **2020**, *39*, 22–27.
4. Thippani, T.; Mandal, S.; Wang, G.; Ramani, V.K. Probing oxygen reduction and oxygen evolution reaction on bifunctional non-precious metal catalyst for metal-air batteries. *RSC Adv.* **2016**, *6*, 71122–71133, doi:10.1039/C6RA13414A.
5. Bian, J.J.; Su, R.; Yao, Y.; Wang, J.; Zhou, J.G.; Li, F.; Wang, Z.L.; Sun, C.W. Mg doped perovskite $LaNiO_3$ nanofibers as an efficient bifunctional catalyst for rechargeable zinc-air batteries. *ACS Appl. Energy Mater.* **2019**, *2*, 923–931.
6. Wang, H.Z.; Xu, W.C.; Richins, S.; Liaw, K.; Yan, L.T.; Zhou, M.; Luo, H.M. Polymer-assisted approach to $LaCo_{1-x}NixO_3$ network nanostructures as bifunctional oxygen electrocatalysts. *Electrochim. Acta* **2019**, *296*, 945–953.
7. Song, W.; Ren, Z.; Chen, S.Y.; Meng, Y.; Biswas, S.; Nandi, P.; Elsen, H.A.; Gao, P.X.; Suib, S.L.*Mater.* Ni and Mn-Promoted Mesoporous Co_3O_4: A Stable Bifunctional Catalyst with Surface Structure Dependent Activity for Oxygen Reduction Reaction and Oxygen Evolution Reaction. *ACS Appl. Mater. Interfaces* **2016**, *8*, 20802–20813, doi:10.1021/acsami.6b06103.
8. Wang, X.; Dou, Y.; Xie, Y.; Wang, J.; Xia, T.; Huo, L.; Zhao, H. A-site cation-ordering layered perovskite $EuBa_{0.5}Sr_{0.5}Co_{2-x}Fe_xO_{5+\delta}$ as highly active and durable electrocatalyst for oxygen evolution reaction. *ACS Omega* **2020**, *5*, 12501–12515.
9. Hansen, K.K.; Electrochemical reduction of oxygen and nitric oxide on Mn-based perovskites with different A-site cations. *Int. J. Electrochem.* **2020**, 4013697.
10. Qu, M.; Ding, X.; Shen, Z.; Cui, M.; Oropeza, F.E.; Gorni, G.; de la Peña O'Shea, V.A.; Li, W.; Qi, D.-S.; Zhang, K.H. Tailoring the Electronic Structures of the La_2NiMnO_6 Double Perovskite as Efficient Bifunctional Oxygen Electrocatalysis. *Chem. Mater.* **2021**, *33*, 2062–2071.
